# Evidence Synthesis of Gene Therapy and Gene Editing from Different Disorders—Implications for Individuals with Rett Syndrome: A Systematic Review

**DOI:** 10.3390/ijms24109023

**Published:** 2023-05-19

**Authors:** Jatinder Singh, Ella Goodman-Vincent, Paramala Santosh

**Affiliations:** 1 Department of Child and Adolescent Psychiatry, Institute of Psychiatry, Psychology and Neuroscience, King’s College London, London SE5 8AF, UK; jatinder.singh@kcl.ac.uk (J.S.); ella.goodman-vincent@kcl.ac.uk (E.G.-V.); 2 Centre for Interventional Paediatric Psychopharmacology and Rare Diseases (CIPPRD), South London and Maudsley NHS Foundation Trust, London SE5 8AZ, UK; 3 Centre for Interventional Paediatric Psychopharmacology (CIPP) Rett Centre, Institute of Psychiatry, Psychology and Neuroscience, King’s College London and South London and Maudsley NHS Foundation Trust, London SE5 8AZ, UK

**Keywords:** gene therapy, gene editing, genetic disorders, neurodevelopment, brain, Rett syndrome

## Abstract

This systematic review and thematic analysis critically evaluated gene therapy trials in amyotrophic lateral sclerosis, haemoglobinopathies, immunodeficiencies, leukodystrophies, lysosomal storage disorders and retinal dystrophies and extrapolated the key clinical findings to individuals with Rett syndrome (RTT). The PRISMA guidelines were used to search six databases during the last decade, followed by a thematic analysis to identify the emerging themes. Thematic analysis across the different disorders revealed four themes: (I) Therapeutic time window of gene therapy; (II) Administration and dosing strategies for gene therapy; (III) Methods of gene therapeutics and (IV) Future areas of clinical interest. Our synthesis of information has further enriched the current clinical evidence base and can assist in optimising gene therapy and gene editing studies in individuals with RTT, but it would also benefit when applied to other disorders. The findings suggest that gene therapies have better outcomes when the brain is not the primary target. Across different disorders, early intervention appears to be more critical, and targeting the pre-symptomatic stage might prevent symptom pathology. Intervention at later stages of disease progression may benefit by helping to clinically stabilise patients and preventing disease-related symptoms from worsening. If gene therapy or editing has the desired outcome, older patients would need concerted rehabilitation efforts to reverse their impairments. The timing of intervention and the administration route would be critical parameters for successful outcomes of gene therapy/editing trials in individuals with RTT. Current approaches also need to overcome the challenges of MeCP2 dosing, genotoxicity, transduction efficiencies and biodistribution.

## 1. Introduction

The molecular landscape and how this may impact the management of individuals with Rett syndrome (RTT) are changing. Rett syndrome can be caused by a pathogenic loss of function of the gene *methyl-CpG binding protein 2* (*MECP2*). This loss of function causes impaired associations with transcriptional machinery, such as reduced binding of *MECP2* to the NCoR/SMRT corepressor complex [1,2], defective assembly of the MeCP2/Rbfox/LASR complex [3] and the failure of the transcription factor 20 (TF-20) complex to interact with *MECP2* on chromatin [4]. This interplay between *MECP2* and transcription factors is likely crucial for normal brain functioning. Impaired chromatin modulation causes the disruption of the methylation of long genes [5] that are critical for the maturation and integrity of neuronal pathways, especially in the developing brain. More recently, a meta-analysis using RNA sequencing has revealed a panel of other gene candidates that could be implicated in RTT pathology, including a chromatin modulator, adenylate cyclase, and a neurotrophin [6]. Looking more broadly, it is likely that *MECP2* has modulatory effects across the genome. Indeed, stress during early life epochs is associated with anxiety and depression, concomitantly reducing MeCP2 expression in healthy females [7]. This finding suggests that the *MECP2* gene has genome-wide influences and that the MeCP2 protein significantly modulates vulnerability to psychopathology. How this vulnerability impacts individuals with RTT remains unclear. However, we have surmised that Emotional, Behavioural and Autonomic Dysregulation (EBAD) underpins the complex nature of symptoms seen in individuals with Rett [8,9], and against a backdrop of *MECP2* mosaicism, this could potentially exacerbate early-life neuropsychiatric illness.

Rett syndrome places a significant burden on families and carers, and there are no transformative therapies. Rather, management of the disorder is primarily focused on symptoms and the associated medical management of co-occurring disorders. Given the critical role of *MECP2* across the genome, it is prudent to explore transformative therapies. In a mouse model of RTT, the neurological defects can be reversed [10], and the disorder can be recapitulated in adult mice [11]. This suggests that molecular therapies could potentially restore neuronal defects and, depending on the molecular therapy used, may offer symptom improvement across the lifespan of the disorder. The feasibility of gene therapies in neurodevelopmental disorders has been described previously [12], and the potential for transformative therapies focusing on RTT has also been discussed in literature [13,14,15,16,17]. Gene therapy is complicated in RTT due to the functional mosaic expression of MeCP2 in the brain cells. Random inactivation of the X chromosome results in cells having impaired MeCP2 and healthy (wild-type) MeCP2. This necessitates delivering the correct amount of genetic information to the brain, as too much genetic information will mimic the symptom pattern seen in *MECP2* duplication syndrome (MDS) [18,19]. Notwithstanding this limitation, recent advances in genome mining using mouse models have partly paved the way to mitigate the deleterious overexpression of *MECP2* [20]. 

When formulating precision medicine therapies for individuals with RTT, it is helpful to have objective biomarkers that can detect changes in the symptom profile. Heart rate variability and electrodermal activity provide a gateway for detecting some of the changes in symptom trajectory as the disorder progresses [21,22]. However, challenges remain. First, how does the developmental trajectory underpin the timing of the intervention? This aspect is critical because we have previously hypothesised in RTT that as the brain develops, transcription factors are probably no longer able to be recruited to methylated checkpoints within neuronal DNA [23], suggesting that the symptoms of EBAD could emerge at different stages as the disorder progresses. Second, how do we select the appropriate administration route for the intervention? 

We can extrapolate useful information from other genetic disorders that affect different organ systems. Despite information being available regarding gene therapy for other disorders, there is a paucity in the literature regarding clinical information that can be extrapolated and put into practise for gene therapy trials in individuals with RTT. We, therefore, wanted to undertake a systematic review of gene therapy across different disorders to see if any useful clinical information could be gathered. Specifically, the review aimed to undertake a systematic review of gene therapy across a range of disorders, see if any themes emerged using a thematic analysis approach and assess whether these themes can be used to inform clinicians, scientists and the wider RTT community when undertaking gene therapy or editing trials in individuals with RTT.

## 2. Methods

### 2.1. Search Strategy

#### Primary Search Strategy

The PRISMA guidelines were used for the systematic review [24]. To achieve this, two authors (JS and EGV) searched the following databases from December 2022: PubMed, Scopus, Cochrane, PsycINFO, Embase and Web of Science. This search was done independently and in a blinded manner. Searches were modified to include truncation symbols (*) to ensure all search terms were covered. Initially, the primary search was focused on capturing any information related to gene therapy and RTT, followed by secondary searches of other disorders that affect brain function, namely Angelman syndrome and MDS. Tuberous Sclerosis Complex (TSC) also shares common features with RTT, with 85% of patients developing Autism Spectrum Disorder (ASD), epilepsy and other shared behavioural problems [25]. Hence, potential gene therapies related to TSC were also searched for. To make the search as expansive as possible, we also searched for gene therapies and other disorders using the methodology described previously [26]. In brief, snowballing was used so that as much of the literature related to gene therapy studies could be traced as possible. The first author (JS) searched independently from the second author (EGV) to reduce the searching bias. Both authors used the PRISMA criteria, and a consensus was reached on the final list of eligible articles to be analysed. The senior author (PS) was consulted if a consensus could not be reached.

### 2.2. Search Terms

Primary Search Terms

The search of the databases used the following keywords: (Rett syndrome OR MECP2) AND (gene therapy*) AND (Brain*)

Secondary Search Terms

(Angelman Syndrome) AND (gene therapy*) AND (Brain*)

(MECP2 duplication syndrome OR MDS) AND (gene therapy*) AND (Brain*)

(Tuberous sclerosis OR tuberous sclerosis complex) AND (gene therapy*) AND (Brain*)

The ‘snowballing’ approach was used to identify additional articles on gene therapy and other disorders.

### 2.3. Population Characteristics (Primary Search)

All records within the databases that reported studies in RTT and other disorders were searched.

### 2.4. Intervention

All records that had information on gene therapy. 

### 2.5. Eligibility Criteria

The following eligibility criteria were used:

Inclusion Criteria

- Full-text records/articles in peer-reviewed academic/scientific journals available electronically from the last 10 years (2012 to present)
- All experiments are conducted on humans or human cells.

Exclusion Criteria

- Articles that were not in English language.
- Studies using animal models.
- Information provided in reviews, preprints, letters to the editor, conference abstracts, book chapters and clinical trial protocols.

### 2.6. Extraction of Data and Thematic Analysis

The extraction of data and thematic analysis were performed as previously described [26]. The first author extracted the data, synthesised the information into a table (Table in Section 3), and performed the manual coding for the thematic analysis. The second author independently reviewed the thematic analysis, and the themes that arose were discussed before reaching a consensus. The final themes that emerged were based on the agreement of all authors. Microsoft 365 running Excel software (2023) was used to display the theme frequencies.

## 3. Results

**Figure 1:**
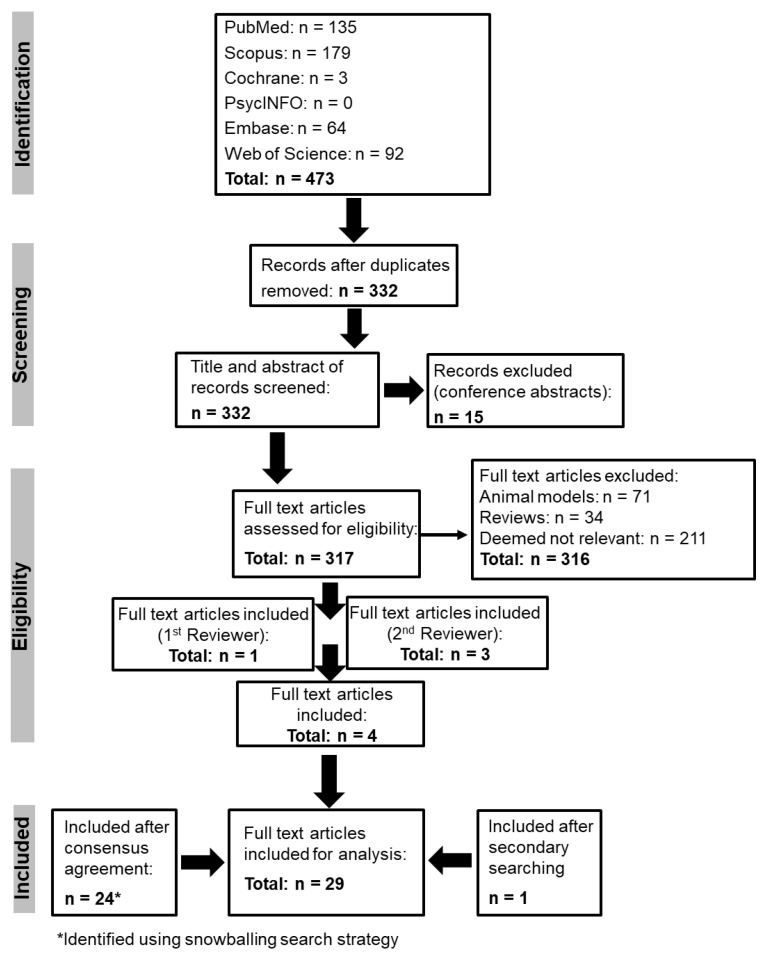
PRISMA flow diagram.

Using the PRISMA (Figure 1) criteria, 473 records were identified, and after the duplicates were removed, 332 records were screened. At the eligibility stage, another 316 articles were removed, and 4 full-text articles were identified. A search of the databases using the secondary search terms identified another article (Supplementary Table S1), and a further 24 articles were identified using the snowballing search strategy. This strategy searches the reference list for relevant articles on gene therapy and gene editing across different disorders. It ensures that as much of the literature as possible can be covered so that further eligible articles may be identified [26]. A total of 29 articles were included in the final analysis, and Table 1 shows the evidence synthesis from the 29 included articles to identify the themes that emerged.

### 3.1. Article Characteristics 

The synthesis of information extracted from the 29 included articles is presented in Table 1 and spanned a range of disorders affecting different organ systems.

#### 3.1.1. Leukodystrophies

Five articles assessed lentivirus vector-mediated gene therapy in patients with leukodystrophies. Three articles covered the use of lentivirus haematopoietic stem cell gene therapy in patients with metachromatic leukodystrophy (MLD) [27,28,29], while two focused on cerebral adrenoleukodystrophy [30,31]. 

#### 3.1.2. Neuromuscular Disorders

The systematic review also captured information on neuromuscular disorders and gene therapy from five articles. Three of these articles explored the safety and efficacy of adeno-associated virus (AAV) gene therapy in individuals with Duchenne muscular dystrophy (DMD) [32], limb-girdle muscular dystrophy type 2D (LGMD2D) [33] and Becker muscular dystrophy [34]. The effectiveness of gene replacement therapy in infants with Spinal Muscular Atrophy Type 1 (SMA1) was also evaluated in two studies [35,36].

#### 3.1.3. Haemoglobinopathies

Gene therapy in haemoglobinopathies was assessed in four studies. A follow-up study of AAV mediated gene therapy in 15 adults with haemophilia A was examined in 1 study [37], while lentivirus vector gene therapy was trialled in 22 individuals aged 12–35 years with transfusion-dependent β-thalassemia in another [38]. Lentivirus-mediated gene therapy has also been used to correct a deletion in the *alpha globin* gene in a patient with sickle cell disease [39]. Another study explored the feasibility of CRISPR-associated protein 9 (Cas9) gene editing technology in haematopoietic stem cells isolated from a patient with Sickle cell disease and a patient with β-thalassemia [40].

#### 3.1.4. Lysosomal Storage Disorders

One article described the use of AAV gene therapy to deliver functional *hexosaminidase A* gene in individuals with infantile Tay–Sachs disease (TSD). In this study, patients were treated with gene therapy at 7 and 30 months of age, and both patients had a confirmed diagnosis of TSD [41]. Another article described the safety and efficacy of intracerebral injection of an AAV encoding genes *N-sulfoglycosamine sulfohydrolase (SGSH)* and *sulfatase-modifying factor* (*SUMF1)* in four children with Mucopolysaccharidosis Type IIIA (MPS Type IIIA) disease [42]. A different approach using antisense oligonucleotides was used in neuronal ceroid lipofuscinoses. This was a proof-of-concept study in a 6-year-old girl with neuronal ceroid lipofuscinosis 7 (CLN7) that used a personalised splice modulating antisense oligonucleotide (Milasen). The antisense oligonucleotide was administered via an intrathecal bolus injection to restore *MFSD8* (Major Facilitator Superfamily Domain Containing 8) gene function [43]. Single-dose lentivirus transduced with *α-gal A* (alpha-galactosidase A) CD34+ gene therapy was also assessed in five adult males with Fabry disease [44]. In this study, all patients had Type 1 (classical) Fabry disease and had received enzyme therapy for at least 6 months prior to study enrolment.

#### 3.1.5. Rett Syndrome

These articles characterised models of gene editing technologies in primary cells derived from individuals with RTT. One study characterised a human neuronal model from RTT-derived fibroblasts and induced Pluripotent Stem Cells (iPSCs)-derived neurons for gene editing of mutation hotspots [45], while another evaluated AAV-mediated gene editing of *FOXG1* variants in patient-derived fibroblasts, iPSCs and iPSC-derived neurons [46]. Induced fibroblasts in twin girls with RTT aged 10 years old were used to understand how MeCP2 is involved in the differentiation of neuronal cells [47].

#### 3.1.6. Angelman Syndrome

One study was identified that evaluated an Angelman syndrome model composed of an *Ube3a (E3 ubiquitin-protein ligase)* gene expressing lentivector in human-derived haemopoietic stem cells [48]. Other gene editing methods have also been used, including Cas9 mediating targeting to unsilence paternal *Ube3a* in primary human neural progenitor-derived cells [49]. 

#### 3.1.7. Retinal Disorders

Two articles examined the safety and efficacy of AAV-mediated retinal pigment epithelium-specific protein 65 kDa (*RPE65*) gene replacement therapy in patients with inherited retinal dystrophies [50,51]. 

#### 3.1.8. Immunodeficiencies

Gene therapy has been used for the management of rare immunodeficiencies in patients. The safety and efficacy of lentivirus haemopoietic stem cell gene therapy has been evaluated in eight children with Wiskott–Aldrich syndrome (WAS) [52]. An earlier study using seven patients with WAS also assessed the safety and efficacy of gene-transduced haemopoietic stem cells [53].

#### 3.1.9. Familial Amyotrophic Lateral Sclerosis

Gene therapy was assessed in two patients with familial amyotrophic lateral sclerosis (ALS). This case study used a single intrathecal infusion of AAV encoding a microRNA targeting the gene encoding *superoxide dismutase 1* (*SOD1*) [54].

#### 3.1.10. Huntington’s Disease 

This randomised, placebo-controlled double-blind multiple ascending dose study evaluated the safety and efficacy of an intrathecally administered antisense oligonucleotide (HTT_Rx_) in patients with Huntington’s disease [55]. The HTT_Rx_ was designed to target and inhibit the huntingtin gene (*HTT*), thus reducing the amount of mutant HTT protein in patients.

### 3.2. Thematic Analysis of the Included Studies

**Figure 2:**
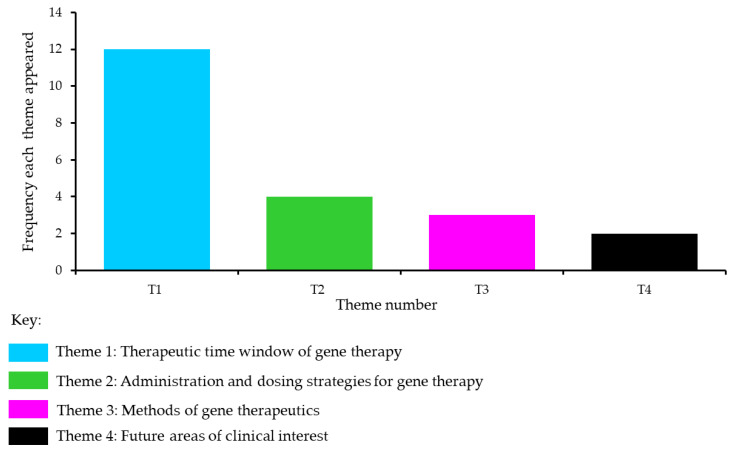
Frequency of the four themes that emerged.

Four themes emerged after extracting the data from the 29 articles. The most prominent theme that emerged was the ‘Therapeutic time window of gene therapy’ and how this could influence the effectiveness of gene therapy and/or gene editing in different disorders. Two other themes about ‘Administration and dosing strategies for gene therapy’ and ‘Methods of gene therapeutics’, also emerged. The theme with the lowest frequency was ‘Future areas of clinical interest’. The frequency of these themes is presented in Figure 2, and the themes are described in the next section. 

#### 3.2.1. Theme 1: Therapeutic Time Window of Gene Therapy 

This theme emerged from 12 articles and ranged across the different disorders, including MLD [27,28], cerebral adrenoleukodystrophy [30,31], SMA1 [35,36], TSD [41], Mucopolysaccharidosis Type IIIA [42], Angelman syndrome [48,49], retinal dystrophy [51] and WAS [52]. Gene therapy using AAV delivered functional *hexosaminidase A* in two patients with infantile TSD [41]. Patients (TSD-001 and TSD-002) were treated at 30 and 7 months of age, respectively. While patient A (TSD-001) remained seizure-free for 5 years and was clinically stable, patient B (TSD-002) deteriorated 6 months post-treatment. Nevertheless, increased myelination in several brain structures and maintenance of corpus callosum volume post-treatment was noted in patients, suggesting a deviation in the natural history of the disease. Despite a scarcity of longitudinal natural history data in TSD, these findings support the view of early treatment in TSD. Further evidence supporting this theme emerged from studies in infants with MLD [27,28]. In MLD, infants who were entering rapid disease progression did not benefit from arsa-cel treatment [27] and it was suggested that the extent of clinical benefit was likely to be associated with the timing of gene therapy and disease onset [28]. This premise is also supported by the study of four males with cerebral adrenoleukodystrophy [30]. In this study, patients were treated with gene therapy between 4.4 and 7.5 years. Neurological deterioration was seen in all patients except for the youngest patient. The authors proposed that this could be because the gene therapy was not initiated early enough for the other patients. Of interest was the finding that in another study of 17 males (median age at study enrolment: 6 years) with cerebral adrenoleukodystrophy, patients who were the most functionally impaired also had the lowest number of lentiviral copy numbers [31].

In Angelman syndrome, the authors suggested that restoring *Ube3A* function during the embryonic and early postnatal periods is likely more effective than restoring gene function during the adult phase [49]. This is also echoed by the findings in infants with SMA1, which propose that for optimal outcomes, the gene therapy intervention should be attempted at the youngest age possible before symptoms emerge [35,36]. Findings from other disorders also support the hypothesis of early intervention. Following intracerebral injection of the AAV encoding genes *SGSH* and *SUMF1* in four children with Mucopolysaccharidosis Type IIIA disease, neurocognitive improvements were observed mainly in the youngest child aged 2 years and 8 months, suggesting that therapy in patients with MPS Type IIIA would be most efficacious for children <5 years of age with no noticeable brain atrophy [42]. In lentivirus haemopoietic stem cell gene therapy in eight children with WAS, disease severity and age at treatment were some of the factors that could account for the differences observed in studies [52]. 

#### 3.2.2. Evidence against a Therapeutic Time Window

In some other studies, the information regarding the clinical benefits of early gene therapy intervention was unclear. In a study assessing an Angelman syndrome model using *Ube3a*-expressing lentivectors in haemopoietic stem cells to deliver functional UBE3A, the authors suggested that their approach is not dependent on a critical time window [48]. Further, in the study assessing the safety and efficacy of AAV gene therapy for functional replacement of the faulty gene *RPE65* in 12 patients with Leber’s Congenital Amaurosis, the greatest improvement was observed in the older patient group ranging from 17 to 23 years of age [51]. Interestingly, functional improvements in retinal sensitivity were lower in younger patients with the most preserved retinal structure. The reason for the weaker effect could be explained by the limited supply of the light-sensitive component (11-*cis* retinal) in relatively preserved photoreceptor cells in younger patients, which may not reach the threshold required for the *RPE65* to produce functional improvement [51].

The included studies also had different gene therapy routes, which will be described in the next section.

#### 3.2.3. Theme 2: Administration and Dosing Strategies for Gene Therapy 

This theme emerged from four studies that described differences in administration routes and dosing strategies. In a randomised controlled trial of four males aged 4–6 years with DMD, high-dose rAAVrh74.MHCK7 micro-dystrophin cassette avoids the need for dose escalation, thereby minimising AAV exposure [32]. Site-directed delivery of AAV may also obviate the need for systemic delivery. This was demonstrated in a study where isolated limb infusion (ILI) delivery of an AAV in patients with LGMD2D specifically targeted to the affected limb replaced the need for widespread gene delivery [33]. In a study of two individuals with familial ALS using a single intrathecal infusion of AAV encoding a microRNA targeting *SOD1*, the authors showed that AAV-targeted gene suppression in ALS offers a way to provide a sustained effect using a single dose [54]. A randomised placebo-controlled double-blind study in patients with Huntington’s disease demonstrated a dose-dependent decrease in the concentration of mutant HTT in the cerebrospinal fluid (CSF) following intrathecal administration of HTT_Rx_ [55].

#### 3.2.4. Theme 3: Methods of Gene Therapeutics

When assessing the studies, three articles described different gene therapeutic methods that may enrich current practises. For example, a proof-of-concept study using an antisense oligonucleotide in a 6-year-old female with CLN7 demonstrated that the method could provide a novel template for the rapid development of personalised corrective treatments in patients with rare genetic conditions within 12 months [43]. Another study developed a gene editing CRISPR/Cas9 toolkit to correct mutations in primary cells derived from individuals with RTT [45]. In this study, the CRISPR/Cas9 method corrected the mutational hotspot (c.473 C > T) with high precision in primary cells derived from individuals with RTT. Another article by the same group showed that AVV-CRISPR/Cas9 technology could target and correct *FOXG1* variants in patient-derived primary cells with high precision [46]. 

#### 3.2.5. Theme 4: Future Areas of Clinical Interest

This theme emerged from two studies that could help optimise gene therapy’s effectiveness in the future. In Becker muscular dystrophy, where a myostatin antagonist was delivered using an AAV, pre-treatment assessments such as MRI could facilitate gene transfer [34]. The host response variability due to post-translational modifications in factor VIII was explored in AAV-directed gene therapy in 15 adults with severe haemophilia A [37]. The study suggested that there was no obvious pattern to explain response variability. However, the authors surmised that glucocorticoid treatment regimens and some other lifestyle factors could play a role in host response variability to gene therapy. Taken together, these studies suggest that treatment regimens and pre-treatment assessments may help optimise gene therapy/editing studies and can be of clinical interest.

#### 3.2.6. Other Considerations

The studies identified in RTT or Angelman syndrome, while important for the development of theme 3, were not specifically clinical trials of gene therapy. Moreover, the systematic review did not identify any records concerning *MECP2* duplication syndrome (MDS) or Tuberous Sclerosis Complex (TSC). Nevertheless, given the overlap in symptoms between RTT, Angelman syndrome, MDS and TCS, it would be prudent to synthesise the key information across these disorders. Small-molecule therapies have been trialled for patients with Angelman syndrome, and one (gaboxadol) showed some improvement in patient symptoms in a Phase 2 study [56] but did not translate to noticeable improvements in a Phase 3 trial [57]. However, Phase 1/2 clinical trials using antisense oligonucleotides are currently recruiting patients with Angelman syndrome [58,59,60]. Lentivector-transduced gene therapy has restored function in a mouse model of Angelman syndrome [48], and other gene replacement therapies are at the pre-clinical stage [57]. 

There is extensive literature on the use of mouse models in RTT and MDS [61]. Previous work has shown that even mild overexpression of MeCP2 can lead to progressive neurological deterioration in mice [62]. Genetic rescue or antisense oligonucleotides can reverse the deleterious phenotypes in *MECP2* duplication mice [63]. While antisense oligonucleotides offer a promising therapy for neurological disorders [64], it is unlikely that animal models can faithfully recapitulate the panoply of symptoms seen in patients with MDS, and further work on genetic manipulation is warranted to increase the effectiveness of first-in-human clinical trials. Lastly, significant advances in the genetics of TSC have been made to engender a precision medicine approach for patients with TSC [65]. A reduction in brain pathology has been observed in a mouse model of TSC2 given intravenous AAV gene therapy [66], and a landmark study that generated a human organoid model for TSC and identified caudal late interneuron progenitor (CLIP) cells [67] has furthered our understanding of brain development in patients with TSC. All this information would be helpful for the next generation of studies on patients with TSC. 

Despite the information and evidence synthesis from the systematic review findings, there is a scarcity in the peer-reviewed literature on gene replacement therapy in individuals with RTT. In 2021, a gene therapy trial in individuals with RTT was terminated [68]. Nevertheless, two clinical trials are in the pipeline. One of these is using miRNA-Responsive Auto-Regulatory Element (miRARE) [20] packaged into an AAV in Rett females ≥18 years of age [69]. The other will be using AAV together with Expression Attenuation via Construct Tuning (EXACT) technology in paediatric individuals with RTT [70]. It is therefore crucial to place the current information learned from the thematic analysis in context for individuals with RTT. This will be discussed in the next section to enrich our current understanding of gene replacement studies in individuals with RTT and other paediatric neurodevelopmental disorders.

## 4. Discussion

The information synthesised from various disorders affecting different organ systems enabled the development of themes. These themes were as follows: (I) the therapeutic time window of gene therapy; (II) administration and dosing strategies for gene therapy, (III) methods of gene therapeutics and (IV) future areas of clinical interest. These themes enrich our current understanding of gene therapy across different disorders and how it may improve future understanding, especially in the paediatric population. The evidence synthesis provides a useful segue into RTT to see if evidence emerging from the themes can be extrapolated to individuals with RTT, particularly from the perspective of timing and administration of gene therapy. Timing and administration of gene therapy or gene editing would be critical factors for improving functional outcomes in RTT, and the next section will attempt to provide additional insight into the following questions:

1. Is there a critical therapeutic time window for gene therapy or gene editing in individuals with Rett syndrome?
2. What is the most appropriate route of administration and dosing strategy?

### 4.1. Is There a Critical Therapeutic Time Window for Gene Therapy or Gene Editing in Individuals with Rett Syndrome?

Of the articles comprising this theme, about 80% (10/12 articles) alluded to the fact that the timing window within which a patient is treated with gene therapy would be a crucial factor when determining the success of gene therapy in the context of halting disease progression and clinical stabilisation. In MLD, a retrospective analysis of baseline characteristics was used to define the most appropriate therapeutic time window [27]. This included patients without cognitive decline before impairments in gross motor skills had developed and the use of additional biomarkers to assist in predicting the treatment response. Rapid disease progression at treatment onset, as shown for some patients with early juvenile MLD, could predict treatment resistance [28]. Some symptoms might also be developmentally resistant to change. For example, in MPS Type IIIA, brain atrophy probably occurs very early on, and therefore, treatment at a later stage may not reverse this atrophy [42]. In Leber’s Congenital Amaurosis, variabilities in response to treatment could be dependent on the timing of the intervention and whether retinal degeneration had already occurred [51].

Some other evidence in SMA has shown that because infants were already symptomatic at enrolment, i.e., irreversible motor milestone loss had already occurred, restorations of motor milestones following gene therapy were not as good as those in neurotypical controls [35]. The clinical severity of SMA, based on the number of copies of *SMN2,* has also initiated the development of treatment algorithms for newly diagnosed infants, with those who are more severe being expedited for treatment sooner [71]. Another approach for treating SMA, such as using the antisense oligonucleotide nusinersen for infantile-onset SMA [72,73], has shown that nusinersen might be more effective in patients with shorter disease duration [74,75]. Other data has demonstrated that nusinersen also shows clinically meaningful improvements in late-onset SMA [76] and adults [77] with established SMA even after 10 months of treatment [78]. While in some disorders, the effectiveness of treatments may span across the age range, the most effective time window of other disorders is uncertain. In TSD, pre-symptomatic intervention is supported because overt neurodegeneration and neuroinflammation were not observed in the transcriptome of human foetal brain samples with confirmed mutations in the *hexosaminidase A* gene [79]. Recent data from a mouse model of Angelman syndrome showed that the early post-natal period would be a critical period for intervention [80], and treating a neuronal circuit defect early on in newborn mice with Huntington’s disease can arrest disease pathology later in adult mice [81]. Other evidence from an Angelman syndrome model using a *Ube3a*-expressing lentivectors in haemopoietic stem cells showed that *Ube3a* function could be restored beyond the typical early post-natal period [48]. Pre-symptomatic training in a mouse model of RTT can reduce functional impairments further and supports genetic screening for newborns with Rett [82]. How these findings compare to human brain development is unknown. The developmental trajectory and the adaptations within the brain that have occurred in individuals with RTT are unclear. It is highly probable that during early developmental epochs, there are time-sensitive windows that alter the neuronal pathways and cause long-lasting changes within the brains of Rett patients. Restoring gene function in RTT might also increase brain size by stimulating neuronal architecture [15]. In this vein, initiating treatment early in development would make more sense when the skull is more malleable. The current evidence synthesis suggests the therapeutic time window could be relatively narrow in RTT. Early gene therapy or gene editing intervention is more critical, especially during the pre-symptomatic stage, as this could prevent symptom pathology. Intervention at later stages in RTT could benefit by helping to clinically stabilise the patient and prevent disease-related complications from worsening. If gene therapy or gene editing could have the desired effect at the genome level, it is highly likely that in older patients, there will need to be a combined rehabilitation effort to reverse the entrenched neurological impairments.

In the UK, every newborn child is offered a heel prick blood spot test that tests for nine conditions ranging from sickle cell disease to inherited metabolic diseases such as phenylketonuria [83]. Recently, a caregiver survey across the UK and the Republic of Ireland assessed the importance of early diagnosis and newborn screening for MLD [84]. In this survey, 95% of caregivers responded that newborn screening was “very or extremely important”, while 86% responded that if MLD was detected at birth “*it would have changed their child’s future*” [84]. These results, from a caregiver perspective, underscore the importance of early diagnosis and newborn screening for MLD [84]. The England Rare Disease Action Plan 2022 is working towards improving how decisions are made on screening for rare disorders in healthy newborns using whole genome sequencing (WGS) [85]. There is support for WGS as a newborn screening tool from participants, as described in a public dialogue commissioned by Genomics England and the UK National Screening Committee (UK NSC) [86]. Mutations of *MECP2* within the population are not homogenous because the mutation has been described in an Angelman syndrome phenotype [87] and some other neuropsychiatric disorders [88]. There is also evidence of patients who have documented disease-causing *MECP2* mutations but do not show the clinical features of RTT [89]. Newborn screening for RTT would also need to consider the alterations of the *MECP2* gene within the healthy population. Reduced gene expression can increase the vulnerability to stress in healthy females [7], and variations in healthy control populations have also been described [90]. Any newborn screening strategy will need to have a threshold at which *MECP2* becomes pathogenic. However, this may not indicate whether the individual will go on to be diagnosed with RTT. It would therefore be crucial to decipher the mutational profile of an individual with mutant *MECP2* before genetic counselling is offered [91].

The decision to include RTT and other diseases in an existing newborn screening programme will ultimately depend on government regulators and ethical review boards. Currently, two gene therapy trials for individuals with RTT are in the pipeline. If the optimal therapeutic window is after birth or shortly after symptoms appear, there will be significant challenges to overcome. Early risk assessment for RTT would probably need to comprise similar schemas adapted for other diseases. In TSC, early risk assessment during pregnancy or the early post-natal period can provide valuable information on epilepsy, ASD, and developmental delay [65]. Pre-symptomatic observations using ultrasound and MRI imaging can include assessing the foetus for TSC-associated central and peripheral lesions [92]. Following the early post-natal period, MRI assessments have been shown to describe anatomical connectivity patterns of the fusiform gyrus to detect the risk of developing ASD in infants with TSC as early as 1 year of age [93]. Further evidence has shown abnormal changes in early white matter development in children with TSC who develop ASD [94]. In another study of 41 children with TSC with a foetal MRI (median gestational age of MRI: 33.3 weeks), higher MRI lesion scores were associated with an ASD diagnosis, and motor and cognitive impairments at 2 years of age [95]. These findings suggest that in TSC, foetal MRI can predict the onset of neuropsychiatric impairments later in life and suggest that biomarkers might help to identify early changes associated with ASD so that interventions can be given as early as possible. Further, in a study of 62 young boys with cerebral adrenoleukodystrophy who received a haematopoietic stem cell transplant (age: mean [SD] at transplant: 8.37 [2.80] years), detection of cerebral disease using MRI at the time of transplant was predictive of longer-term neurological outcomes [96].

Unlike TSC, where there are apparent neuroanatomical signs, whether this approach would be feasible for individuals with RTT is unclear. In a study of 28 girls with RTT (mean age (years) ± SD: 3.5 ± 1.25 years), MRI revealed structural brain abnormalities such as decreased grey matter in the insula, frontal cortex and limbic regions [97]. Diffusion MRI of Rett brains may also allow for finer details of brain microstructure to be revealed at various stages of development [98]. However, the modifications in brain architecture seen during MRI probably represent an epoch in RTT where changes are irreversible and would not be of benefit for pre-symptomatic screening. At present, the lack of multimodal MRI data coupled with developmental models during the very early stages of RTT [99] hinders biomarker development and precludes clinically meaningful inferences from being drawn. 

The development of clinical biomarkers of RTT at very early developmental stages is warranted to assess the effectiveness of gene therapy or gene editing and predict side effects. Treatment at a very early developmental milestone could also trigger unexpected treatment-related effects unique to this age group. Gene therapy or gene editing in children may also alter neuronal architecture, which could confer neuronal vulnerability beyond the therapeutic window [100,101]. Nevertheless, despite these concerns, gene therapy or gene editing in RTT offers hope for further transformative therapies, and in the next section, administration and dosing strategies will be discussed.

### 4.2. What Is the Most Appropriate Route of Administration and Dosing Strategy?

The evidence synthesis enabled further understanding of administration and dosing strategies across different disorders. In TSD, where intrathecal or thalamic delivery methods were used, hexosaminidase A levels in the CSF may not necessarily reflect brain tissue activity. Indeed, protein levels in the CSF should not be used as an index for penetration into the brain [102]. Thalamic delivery could result in different protein expression levels across brain regions [41]. Similarly, while a dose-dependent decrease in mutant HTT protein was observed in the CSF of patients with Huntington’s disease, there was no change in functional, neurological, psychiatric or cognitive outcomes in patients who received placebo versus those receiving active treatment [55]. One reason could be that the oligonucleotide therapy did not reach the correct parts of the brain in patients with Huntington’s disease. In SMA, AVXS-101 was engineered with a strong and continuous promoter to provide high and long-lasting protein expression levels [35]. One of the challenging obstacles when delivering genetic material to the brain is overcoming the blood-brain barrier (BBB) [103]. Different strategies have been explored to circumvent this problem [102]. Non-invasive magnetic resonance-guided focused ultrasound can open the BBB, as demonstrated in five patients with mild to moderate Alzheimer’s [104]. This method was also used to selectively target the BBB to open the parieto-occipito-temporal junction in patients with Parkinson’s disease with dementia [105]. Stereotactic intracerebroventricular injection approaches could maximise vector exposure to key brain regions and help lower the dose needed. However, these methods would only be practical in Rett patients if brain regions more amenable to gene therapy or gene editing could be identified. Gene delivery via the vagus nerve is also an attractive option, as it offers a conduit of access for the ANS’s central and peripheral components. This method might be more useful for targeting autonomic dysregulation but not when targeting other brain areas, such as those involved in motor function. If autonomic dysregulation was targeted, the timing of vagus nerve-mediated gene delivery would need to adjust depending on age. The vagus nerve is partially myelinated at birth, with the highest increase being between 30–32 weeks (gestational age) and 6 months post-birth [106], and this myelination continues through adolescence [107]. In animal models of RTT, there is impaired expression of myelin-related proteins [108] and dysregulated white matter myelination [109]. Post-mortem data from the brains of Rett patients suggest lower levels of MeCP2 in glial cells [110], dysregulated myelination in the cerebellum, and global impairments of genes associated with myelination in patients [111]. These findings may not be unique to RTT, and abnormal myelination profiles have been noted for other neurodevelopmental disorders [112]. These studies point towards abnormal myelination, and it is unknown how effective gene delivery using vagus bathing will be in individuals with RTT, but it is probably more relevant to use this approach during early life epochs.

Current gene therapy trials in RTT use AAV delivery vectors. However, even AAV9 has modest BBB permeability [103] and needs to be modified to increase its penetrance and neuronal tropism when administered systemically [25]. In other instances, where some cell types might be refractory to current AAV gene delivery methods, AAV can be modified with novel capsids with preferential tropism to specific cell lineages, for example, targeting oligodendrocytes in white matter disease [113]. The literature regarding AAV9 and gene replacement methods in animal models of RTT is extensive and has been reviewed recently [13]; however, there are some additional important points to consider. In 2017, AAV capsids were developed that could increase their transduction efficiency within the Autonomic Nervous System (ANS) when delivered systemically [114], and this approach was used to increase the transduction efficiency of a transgene cassette in a mouse model of RTT [115].

One of the drawbacks of using AAVs as delivery vectors is the amount of information they can carry. Using CRISPR with viral vectors can work around this limitation, as shown in the Angelman syndrome model [48]. It was also used to correct a mutation hotspot in primary cells derived from individuals with RTT [45]. A previous study has also shown that the CRISPR/Cas9 methods could repair mutations with high efficiency in human induced pluripotent stem cells [116]. The CRISPR method provides an efficient way to integrate larger amounts of genetic material into cellular genomes [117]. In RTT, gene therapy trials use modified versions of AAVs with proprietary technology to optimise AAV size constraints and regulate the dosage of *MECP2* delivered so that expression levels can be tightly controlled [69,70]. The bidirectional sensitivity of *MECP2* shares features with other dosage-sensitive genes [118] and may regulate other dosage-sensitive genes critical for brain function [119]. Evidence from mouse models suggests that the expression window to cause deleterious changes is very narrow. In RTT, this is critical because, in females, brain structures will have neurons expressing a normal X chromosome while other neurons will express an aberrant chromosome. Expression of *MECP2* to cause pathogenic symptoms of MDS in mouse models of RTT is between 1.6 and 2.4 times the normal level [25,120,121] and about a 3.8-fold overexpression is lethal [120]. Too much of an AAV vector expressing *MeCP2* can cause off-target effects in a female mouse model of RTT [122]. More recent evidence has shown that motor pathways, particularly striatal circuits, could be susceptible to MeCP2 levels [123]. 

In summary, current gene therapy trials in individuals with RTT rely on gene delivery methods based on modified AAV with embedded technologies to regulate dosage levels of *MECP2* [69,70]. There are caveats concerning administration routes, immune responses, transduction efficiencies and regulating the MeCP2 level in cells. In animal models, most of the symptoms of RTT improve. However, it is unknown whether this will be replicated in clinical trials. Extrapolating the biodistribution of delivery vehicles from animal models is also a concern. There are limitations in the evidence base concerning the biodistribution of different CSF routes and whether particular regions could be more easily transduced [124]. In 2021, a gene therapy trial in individuals with RTT was terminated. Here, poor biodistribution in preclinical studies on non-human primates could not sufficiently warrant a transition into human patient trials [125]. However, animal models of RTT have significantly improved our understanding of this area, and the current clinical trials provide a solid platform for building upon and understanding gene therapy/editing in other rare neurodevelopmental disorders. 

## 5. Conclusions

As far as we know, this is the first study that combined a systematic review with a qualitative thematic analysis to critically evaluate gene therapy across different disorders ranging from ALS to haemoglobinopathies, immunodeficiencies, leukodystrophies, lysosomal storage disorders and retinal dystrophies. Disorders that show symptom overlap with RTT, such as Angelman syndrome, MDS and TSC, were also analysed. The emerging themes allowed the information to be extrapolated to RTT and indicated that the timing of gene therapy and the administration route would be critical parameters for successful outcomes. Gene therapy for disorders with one organ system implicated has fared better when compared to disorders with a more diffuse target. Those that have improved the patient’s quality of life are evidenced by studies done in neuromuscular disorders, haemoglobinopathies, retinal disorders and immunodeficiencies where there has been a more focused target. Where the target is more diffuse, the pattern is not as obvious. Gene therapy has been shown to be effective in children with MLD but not for other disorders such as Huntington’s and RTT, where several organ systems are implicated, and the brain is the primary target. Table 2 provides a focus on disorders covered with more similar characteristics to RTT, i.e., the brain as the primary target and key points for consideration. Our evidence synthesis suggests that the timing of gene therapy/editing would be a predictor of better response. Those patients without much neurological and motor deterioration will have the most benefit. Lower doses of genetic products could be used if treatment is initiated earlier, and intracranial administration routes might be more effective. Finally, there should be a continued focus on research to better understand if certain brain regions respond better to gene therapy/editing.

**Figure 3:**
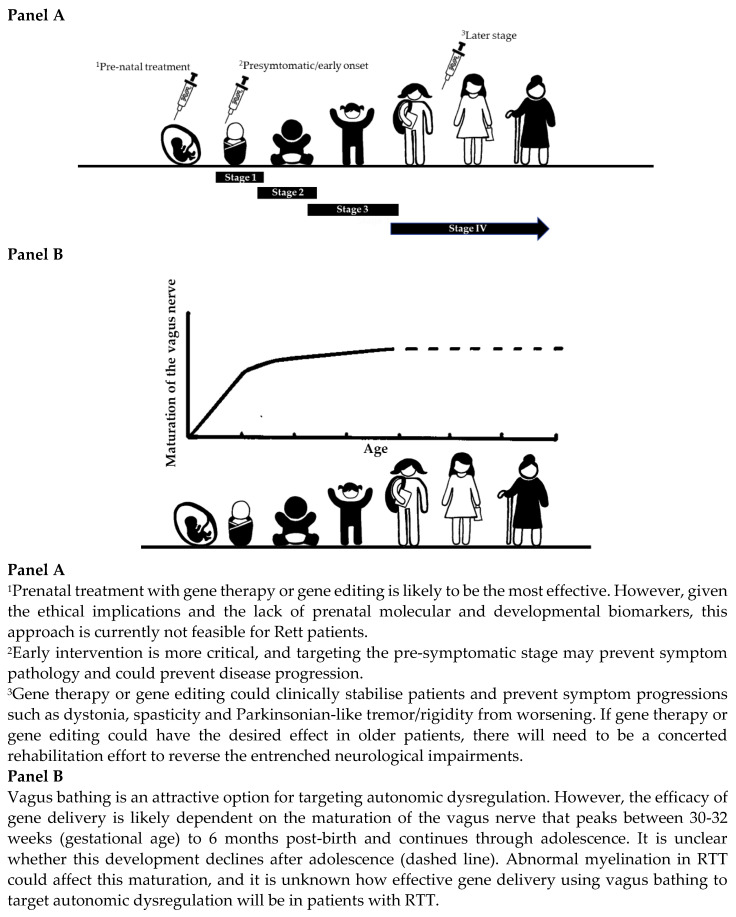
Gene therapy/gene editing approaches in Rett Syndrome.

In RTT, gene therapy or gene editing can either improve impairments or clinically stabilise them, i.e., prevent symptoms from worsening during the lifespan of the disease. Gene therapy or gene editing would probably be optimal before Stage 1 of RTT but should be avoided during the regression phase (Stage 2). The regression phase is characterised by worsening symptoms, and treatment at this stage could potentially mask gene therapy or gene editing mediated side effects. Gene therapy or gene editing may still benefit past the plateau stage (Stage 3) for clinical stabilisation, such as preventing the motor decline from worsening. It is unknown whether neurogenesis would occur following gene therapy or gene editing during the later stages (Stage 4) and whether the extent of neurogenesis would differ depending on when the genetic manipulation had occurred. However, challenges remain. There are no obvious molecular or developmental biomarkers that would assist in unravelling the crucial development epochs of RTT before or at the early onset before regression occurs. Our synthesis of information has further enriched the current knowledge base, which, when combined with other evidence, can be used to optimise gene replacement in individuals with RTT (Figure 3) but can also be applied to other neurodevelopmental disorders. The prevalence of individuals with RTT is 5 to 10 cases per 100,000 females [126]. This information may assist in decision making when recruiting for gene therapy/editing trials.

The evidence synthesis covers gene therapy and other gene editing approaches such as CRISPR/Cas9. However, X-chromosome reactivation and RNA editing methods could also be viable options for treating individuals with RTT [13,61,127]. Recently, RNA editing of MeCP*2* expression levels in the brainstem has been shown to alleviate symptoms in a mouse model of RTT [128] and epigenome editing was used to reactivate silenced *MECP2* on the inactive X chromosome in RTT-derived stem cells and neurons [129]. Other X-chromosome reactivation strategies are currently in the clinical trial pipeline [130]. Both offer promising alternative therapeutic strategies. While there are advantages and disadvantages to the different methods for treating RTT [127], there will be no perfect solution. Different molecules are being explored in RTT [131,132], and any treatment will likely need to combine genetic-based therapeutic approaches with molecules to target neuronal pathways affected by RTT. 

### 5.1. Future Directions

In England, UK, NICE (National Institute for Health and Care Excellence) has recommended the use of Zolgensma^TM^ (onasemnogene abeparvovec) in children < 6–12 months of age with SMA [133], Libmeldy^TM^ (atidarsagene autotemcel) for children with late infantile or early juvenile type MLD [134] and Upstaza (eladocagene exuparvovec) in individuals aged ≥18 months for the treatment of aromatic L-amino acid decarboxylase (AADC) deficiency [135]. Specific recommendations have also been made for nusinersen for treating SMA [136]. In RTT, gene therapy approaches must overcome challenges concerning MeCP2 dosing, immune responses, transduction efficiencies and biodistribution. Regulating the expression of *MECP2* once gene therapy is initiated in patients is of particular concern, and it would be prudent to consider alternative strategies for brain circuit disorders, such as RTT [137]. Since neuronal cells within brain regions would have wild-type and mutated cells, maintaining neuronal equilibrium would be critical once gene therapy is started in Rett patients. An on-demand cell-autonomous gene therapy based on a modified CRISPR activation method [137] may be particularly suited for RTT because it can potentially normalise neuronal hyperactivity among healthy and deceased circuits. A pharmacogenomic-guided strategy could also assist in minimising drug reactions [138] especially given that patients would need to be on various drugs, such as immunosuppressants, during and after the trial. Long-term immunosuppressant use and potential immune responses caused by viral vectors and transgene products would be important elements to monitor. The timing and duration of said immunosuppressant treatment regimen could also impact the efficacy of AAV-based interventions [139,140]. Pharmacogenomics would also help predict the treatment of non-responders. Treatment non-response is associated with EBAD in Rett patients [9]. It is sensible to target autonomic dysregulation (I) for risk stratification, (II) to identify treatment non-responders, and (III) to capture symptom changes longitudinally. Wearable sensors can be used as objective biomarkers to monitor symptom change in individuals with RTT [21,22,141]. When used with the Multi-system Profile of Symptoms Scale (MPSS) [142] and natural history data from patients within the CIPP Rett Centre, wearable sensors can assist in identifying neurobehavioral profiles and treatment non-responders before gene therapy or gene editing is initiated and afterwards to monitor symptoms. If a specific area, such as autonomic dysregulation, was targeted using gene therapy or gene editing, objective biomarkers could help stratify patients so that those with more severe autonomic dysregulation profiles could have the first exposure to gene therapy. Longitudinal monitoring of symptoms using objective biomarkers would be especially necessary to assess the longer-term health economic impact following gene therapy/editing. An increase in brain size following treatment during early life epochs is another objective biomarker to consider.

### 5.2. Limitations

The literature regarding gene therapy trials is exhaustive. From 2010 to 2020, there were 283 clinical trials done for genetic diseases, with metabolic diseases being the most frequent area targeted by gene therapy [143]. Furthermore, during this time span, there have been about 1700 viral vector-associated gene therapy trials [125]. While extensive, the current evidence synthesis was limited by not being able to trace all the literature on gene therapy or gene editing trials in rare disorders and did not include gene therapy or gene editing studies relating to cancer, on which most of the research has focused during the last decade [143]. Therefore, the present findings should be treated with caution. Even though the evidence synthesis focused mainly on genetic disorders, it is limited in its scope when viewed across other therapeutic areas. It is also difficult to compare outcomes across disorders, particularly when comparing disorders that affect one organ system to those affecting multiple organ systems. The authors acknowledge the limitations of the present evidence synthesis, which used a combined snowballing and systematic review methodology. However, our search strategy and qualitative thematic analysis were comprehensive. Two authors independently reviewed the literature to minimise search bias. The thematic analysis and all articles included were based on a consensus agreement with all the authors. 

## Figures and Tables

**Table 1:** Summarised information from the eligible studies.

Source	Characteristics/Demographics	Clinical Characteristics	Assessment Methods	Relevant Information
[27] Fumagalli et al. (2022)	Long-term follow up of lentivirus haematopoietic stem cell gene therapy in patients with metachromatic leukodystrophy Intention to treat set: Late infantile (n = 16): aged (months ± SD) 12.81 (4.3) Early juvenile (n = 13): aged (months ± SD) 65.86 (33.4) Natural history cohort (untreated control group): Late infantile (n = 19): aged (months ± SD) 20.64 (4.7) Early juvenile (n = 12): aged (months ± SD) 51.98 (19.2)	Study patients had a confirmed diagnosis with pre-symptomatic or early symptomatic metachromatic leukodystrophy (MLD) Patients were treated with arsa-cel^a^ Median follow-up: 3.16 years	Primary endpoints included: Improvements (>10%) in (i) total scores of the gross motor function measure (GMFM-88) at 2 years after treatment and (ii) change in baseline of total peripheral blood mononuclear cell ARSA activity after 2 years	Of the 29 patients treated with arsa-cel, 2 patients died because of MLD progression and another patient due to an ischaemic stroke 13.6 months post treatment. These events were deemed not related to treatment. The gene therapy was well tolerated with no treatment emergent serious adverse events. Patients treated with arsa-cel demonstrated high engraftment of corrected cells with concomitant increased ARSA activity in blood and cerebrospinal fluid. In children with early-onset MLD, treatment with arsa-cel resulted in clinically meaningful and sustained improvements in cognitive and motor function. In addition, treatment also reduced demyelination and brain atrophy when compared to the natural progression of the disease. The findings suggested that patients who were entering rapid MLD progression did not benefit from arsa-cel treatment. The authors suggest that patients with MLD should be diagnosed and treated as soon as possible and underscore the urgent requirement for newborn screening tests for MLD.
[28] Sessa et al. (2016)	Study assessing lentivirus haemopoietic stem cell gene therapy in nine children with MLD Age at gene therapy was between 7 and 59 months	Six patients had late-infantile metachromatic leukodystrophy, two had early juvenile and one had early-onset metachromatic leukodystrophy Post-treatment median follow up was 36 months	Primary endpoints were safety, tolerability and efficacy Efficacy measures were improvements in gross motor function and ARSA activity 24 months post-treatment Effect of treatment on central nervous system myelination was assessed by MRI	No serious adverse events related to the gene therapy was reported. The findings from the study showed that there was sustained and stable levels of gene corrected haemopoietic stem cells and ARSA activity. Seven patients that had received treatment were pre-symptomatic when compared to historical controls with early onset metachromatic leukodystrophy. Gross motor function in six patients at the last follow-up was similar to typically developed children. The authors suggested that the degree of clinical benefit was likely to be due to the time between gene therapy and disease onset.
[29] Biffi et al. (2013)	Study of lentivirus directed gene therapy in three patients with early onset metachromatic leukodystrophy Follow-up period was up to 18 to 24 months post-treatment	All three patients were pre-symptomatic with biochemical and molecular confirmation of *ARSA* mutations Treatment of patients began from 2 to 12 months before disease onset as reported in their siblings Disease evolution was compared with older siblings that had the same ARSA mutational profile and a historical cohort of late infantile metachromatic leukodystrophy	Assessments included neurological and motor evaluation using clinical assessment and gross motor function measure Neuropsychological evaluation was performed using the Bayley Scale for Infant and Toddler Development and sensory motor conduction of nerves was also determined Other assessment of disease progression was done using MRI	There was robust gene expression in the myeloid cells (40–80%). The disease did not progress in the patients when compared to mutation matched siblings. There were also detectable levels of ARSA activity in the cerebrospinal fluid. This indicates that the transduced myeloid cells were also able to deliver active levels of ARSA to the central nervous system of patients.
[30] Bougnères et al. (2021)	Follow-up of lentivirus vector (CG1711 hALD) haemopoietic stem cell gene therapy in four boys with cerebral adrenoleukodystrophy Patient age at diagnoses were 3.5 years (patient 1), 6.3 years (patient 2), 3.6 years (patient 3) and 6 years (patient 4)	The median follow-up period was 8.8 years post-gene therapy Patients received gene therapy at the following age: patient 1 (7 years), patient 2 (7.5 years), patient 3 (4.4 years) and patient 4 (7 years)	Neurocognitive assessments Major functional disabilities assessment MRI to quantify demyelination Vector copy number in peripheral blood cells	The study findings showed that while patient 3 was clinically stable until 8.3 years of follow-up, patients 1, 2 and 4 had significant neurocognitive degradation around 9, 28 and 60 months post gene therapy. There were no adverse events. Myeloid and lymphoid cells expressing the ALD protein decreased by 50% by 5 years and then remained stable at 5–10%. The authors suggest that the neurological deterioration seen in three patients could be due to the low level of transduced microglial cells or the gene therapy was not initiated early enough except for patient 3 that had near normal neurological status before receiving gene therapy.
[31] Eichler et al. (2017)	Lentiviral vector (Lenti-D) mediated haemopoietic stem cell gene therapy in 17 males with cerebral adrenoleukodystrophy Median age (year, range) at enrolment was 6 years (4–13) years Median follow-up (months; range) was 29.4 months (21.6–42.0)	In total, 18 patients were enrolled (1 patient not meeting the eligibility criteria) Eligibility was restricted to those patients demonstrating early signs of the disease	The primary study endpoint was remaining alive and having no significant functional disability 24 months post gene therapy A neurological function scale specific for cerebral adrenoleukodystrophy was used to assess neurological dysfunction	All patients had protein expression and of the 17 that received gene therapy, 88% (15/17) were alive and had no significant functional disability. One patient died from disease progression, and another withdrew and died from transplant-related issues. Most patients were clinically stable with scores on the neurological function scale ranging from 0 to 2 and had limited progression of disease progression on brain MRI when compared to known rates in untreated group. Level of gene expression appears to be critical to prevent neurological deterioration. Although not statistically validated, those patients who were the most functionally impaired had lower numbers of lentiviral copy numbers.
[32] Mendell et al. (2020)	Randomized controlled trial of 4 patients (boys) aged 6, 5 and 4 years	All patients had a confirmed mutation for Duchenne muscular dystrophy (DMD) All received rAAVrh74.MHCK7. micro-dystrophin single high-dose cassette	The 17 item North Star Ambulatory Assessment (NSAA) was used to assess muscle function Serum creatine kinase (CK) levels Other functional assessments including the time-to rise, 4-stair climb, 100 m timed test and dynamometry for knee extensors and elbow flexors	The AAV mediated micro-dystrophin gene transfer was safe and well tolerated with mild (62%) or moderate (38%) treatment emergent adverse events. There were no serious treatment emergent adverse events. Gene transfer was associated with good expression levels and correct localization of functional micro-dystrophin protein. The findings also showed reduced change from baseline in serum CK levels (baseline CK (mean; SD): 27064 (6340.5) U/L versus treatment CK (mean; SD): 8035 (3312.5) U/L). All four patients showed improvements in NSAA levels from baseline and for a 1-year post-treatment. High-dose rAAVrh74.MHCK7 micro-dystrophin cassette avoids the need for dose escalation thereby minimising AAV exposure. Biological markers can be successfully utilised to monitor functional outcomes in gene therapy.
[33] Mendell et al. (2019)	Isolated limb infusion (ILI) delivery at ascending doses of an AAV (scAAVrh74.tMCK.hSGCA) in six patients aged between 8 and 49 years with limb-girdle muscular dystrophy type 2D (LGMD2D)	Safety and tolerability were initially assessed in an adult (aged 49 years) before being evaluated in children (8–13 years) The rationale for ILI-AAV gene transfer was to specifically target the affected limb	The primary assessment measure was the six-minute walk test Secondary measures included a test for muscle strength in knee extensors Alpha-sarcoglycan (*SGCA*) gene expression at 6 months	The study showed that ILI-AAV mediated gene transfer was well tolerated. Using ILI-AAV that is targeted to the affected extremity replaces the need for systemic gene delivery that would require a higher viral load and is more expensive. There was local improvement of the targeted muscle and gene transfer produced SGCA protein at low levels.
[34] Mendell et al. (2015)	Gene therapy for Becker muscular dystrophy Follistatin (myostatin antagonist) was delivered using an AAV (AAV1.CMV.FS344) Patients were separated into two cohorts according to the dose of gene therapy: - Cohort 1: low dose (age: 30, 35 and 37 years, n = 3) - Cohort 2: high dose (age: 24, 30 and 34 years, n = 3)	Gene therapy was administered in a dose ascending regimen to six patients via intramuscular quadriceps injection Patients in cohort 1 and 2 were ambulatory and diagnosed with gene mutations for Becker muscular dystrophy	The primary outcome measure was the 6MWT Secondary outcome assessment was knee extension Monitoring of adverse events	There was some improvement in the distance walked in the 6MWT following gene therapy administration. Two patients were noted as not improved in the 6MWT. Noticeable muscle histology also showed signs of improvement. The authors also suggest that muscle fibrosis might hamper gene expression and related improvement. Future studies that use MRI for pre-treatment assessment and to guide gene transfer could help to facilitate gene expression.
[35] Al-Zaidy et al. (2019)	Effectiveness of AVXS-101 gene replacement therapy in 12 infants with Spinal Muscular Atrophy Type 1 (SMA1) Three cohorts: AVXS-101 treated (n = 12), age (mean ± SD) at first visit: 2.9 months ± 2 Natural history (n = 16), age (mean ± SD) at first visit: 4.0 months ± 2 Healthy controls (n = 27), age (mean ± SD) at first visit: 3.4 months ± 2	Patients received a therapeutic dose of AVXS-101 and compared to age and gender matched cohorts of a natural history SMA 1 cohort and healthy controls	Outcome measures included event free survival, CHOP-INTEND scores, motor milestones muscle action potentials (CMAP) and adverse events	Overall, the study showed that the survival probability, motor function and motor milestone achievement was higher in SMA1 infants treated with AVXS-101. The number of infants treated with AVXS-101 who survived by 24 months was greater than the SMA1 natural history cohort. There were 275 adverse events in the AVXS-101 treatment cohort mostly related to the natural course of the disease. The most frequent adverse event was upper respiratory tract infection seen in 83% of the AVXS-101 treatment group. Further, 53 were serious and of these 2 were said to be treatment emergent. Infants in the AVXS-101 SMA1 cohort were symptomatic at enrolment. The authors indicated that because some irreversible motor neuron loss had already occurred, this cohort did not progress as quickly regarding motor milestones when compared to the healthy infant controls. The authors suggest that for optimal outcomes, AVXS-101 intervention should be attempted at the youngest age possible before symptoms arise.
[36] Mendell et al. (2017)	Study assessing AAV gene therapy for functional replacement of the faulty gene *SMN1* in 15 patients with SMA1 Patients were separated into two cohorts according to the dose of gene therapy: - Cohort 1: low dose (mean age: 6.3 months, n = 3) - Cohort 2: high dose (mean age: 3.4 months, n = 12)	All patients had a confirmed diagnosis of SMA1	The primary outcome measure was safety and tolerability of the gene therapy Secondary outcome was the time until death or whether permanent ventilation was required Exploratory endpoints included comparison of CHOP INTEND motor function ratings within the two cohorts and motor milestones scores between the high dose cohort and those in studies examining disease natural history	The study showed that in patients who received the AAV gene therapy containing the *SMN* gene, survived longer, had better motor milestones and function when compared to the natural history cohort. All patients survived longer than 20 months. Here, 56 serious adverse events were noted in 13 patients in the 2 cohorts of which 2 were rated as treatment emergent grade 4. These were due to elevations in liver enzymes. There were 241 adverse events deemed not serious of which 3 were treatment related. The authors suggest that all patients continued to have improvements in motor function and underscore the premise for early treatment and screening for SMA.
[37] Pasi et al. (2020)	AAV (AAV5-hFVIII-SQ) mediated gene therapy follow-up in adults with haemophilia A	Patients were males (n = 15) with severe haemophilia A and received a single infusion of AAV5-hFVIII-SQ at different dose levels	Parameters such as factor VIII levels, rate of bleeding events, safety and tolerability were followed for up to 3 years	Clinically meaningful improvements were seen in patients administered the AAV5-hFVIII-SQ vector as evidenced by reduction in bleeding rates and patients no longer needing prophylactic factor VIII. To explore host response variability (that might be caused by post-translational modifications in factor VIII), patient profiles and medical records were assessed to provide information on the causality of variability in factor VIII expression. The findings showed while there was no obvious trend in mechanisms responsible for response variability, glucocorticoid treatment regimens could play a role. Some other lifestyle factors may also contribute to response variability.
[38] Thompson et al. (2018)	Study evaluating the safety and efficacy of lentivirus vector (BB305) mediated gene therapy in patients with transfusion dependent β-thalassemia The β-thalassemia cohort consisted of 22 patients aged between 12 and 35 years treated with gene therapy and followed up for 2 years	Patients with any genotype for β-thalassemia and had at least eight transfusions or 100 mL/kg of body weight packed red cells in the last 24 months were eligible for study enrolment	Assessment included monitoring of adverse events, lentivirus integration, levels of total haemoglobin and lentivirus marked β-globin	The findings from the study showed that lentivirus gene therapy decreased or eliminated the requirement of transfusions in the 22 patients. No serious adverse events were reported. The study also indicated that clinical outcomes tended to vary depending on patient genotype.
[39] Ribeil et al. (2017)	Case report of lentivirus mediated gene therapy bone marrow transplantation in a patient with sickle cell disease Patient was 13 years of age at enrolment	The patient had a single-gene deletion in the *alpha globin* gene There were on average 1.6 sickle cell disease events in the 9 years before prophylactic red-cell transfusion	Outcomes were safety including MRI, changes in sickle cell disease related measures, engraftment and gene expression levels	Post 15 months after gene therapy transplant no disease related sickle cell events or hospitalisation occurred in the patient. Medications were also discontinued. The level of anti-sickling beta globin was sustained at 15 months (around 50%). There were no adverse events that were deemed to be related to the transduced cells.
[40] Wu et al. (2019)	Study assessing gene editing in haemopoietic stem cells	Hematopoietic stem cells were isolated from a patient with Sickle cell disease and a patient with β-thalassemia	Assessment of Cas9 gene editing technology in human haematopoietic stem cells Methods such as microscopy analysis and flow cytometry were also used	Edited haematopoietic stem cells from a patient with sickle cell resisted sickling and expressed robust levels of fetal haemoglobin. Moreover, cells from a patient with β-thalassemia had restored globin chains. The methodology could be used for the genetic treatment of other blood disorders.
[41] Flotte et al. (2022)	AAV mediated gene therapy used to deliver functional hexosaminidase A in two patients with infantile Tay–Sachs disease (TSD) Patient A (TSD-001) was treated at 30 months and Patient B (TSD-002) at 7 months	Both patients had a confirmed diagnosis of infantile TSD The vectors were administered via thalamic and CSF delivery Patient A (TSD-001) was given the vector intrathecally due to severe thalamic degeneration while patient B (TSD-002) received both thalamic and intrathecal injections Patient B was administered a lower dose	Primary endpoint was safety following vector administration Additional assessments included immune suppression and responses, enzyme activity in the CSF, MRI, neurological and neurodevelopmental outcomes	There were no vector-related treatment emergent adverse events. In both patients, there was a modest increase in hexosaminidase A detected in the CSF. Patient A (TSD-001) remained seizure free at 5 years of age, while Patient B (TSD-002) showed clinical stabilisation 3 months after treatment, disease progression was noted 6 months post treatment. Increased myelination in several brain structures were noted for TSD-002, which reflects clinical stabilisation when compared to the natural history of the disease and underscores the importance of early treatment.
[42] Tardieu et al. (2014)	Study of intracerebral injection of AAV encoding genes *SGSH* and *SUMF1* in four children with Mucopolysaccharidosis Type IIIA disease followed up for 1 year Patient ages were 6 years 7 months (patient 1), 6 years 5 months (patient 2), 5 years 10 months (patient 3) and 2 years and 8 months (patient 4)	Neurocognitive functioning was better for patient 4 when compared to patients 1–3 who already had reductions in neurocognitive abilities Patients 1–3 had residual enzyme activity; however, no enzyme activity was detected in patient 4 Cerebral atrophy on MRI was noted for patients 1–3. This atrophy was absent in patient 4	Primary endpoints were safety and tolerability Secondary outcome assessments were initial evaluations of efficacy such as changes in brain atrophy alongside changes in the performance of neuropsychological evaluations for each child	Brain atrophy was clinically stable for patients 1 and 3 but increased for the other two patients (patient 2 and 4). A modest improvement was observed in behaviour, attention, and sleep in patients 1–3. In total, 58 mild to moderate adverse events were reported with upper respiratory tract infection as the most common. Neurocognitive improvements were mostly observed in the youngest child (patient 4). This might suggest that therapy would be the most effective for children less than 5 years of age with no noticeable brain atrophy on MRI.
[43] Kim et al. (2019)	Proof of concept (n = 1) study in a 6-year girl with neuronal ceroid lipofuscinosis 7 (CLN7)	Antisense oligonucleotide (Milasen) was personalised to this patient within a year Milasen was administered via an intrathecal bolus injection	Neurological and neuropsychological assessments including the Vineland Adaptive Behaviour Scales, assessments for global motor function and seizure frequency	Milasen had a favourable side effect profile and also led to the reduction in the frequency and duration of seizures. The design was tailored to the patient’s specific mutation especially those that have mutations that can be targeted using mRNA splice-switching. Patients with large deletions also developed earlier onset of hand stereotypies, epilepsy, and scoliosis. This proof of concept offers a route for rapid development of personalised treatments in patients with a rare genetic disease.
[44] Khan et al. (2021)	Single-dose lentivirus transduced with *α-gal A* CD34+ gene therapy in five adult males with Fabry disease Seven patients were enrolled but two failed screening. Age range of the five patients was 29–48 years	All patients had Type 1 (classical) Fabry disease had received enzyme therapy for at least 6 months pre-study enrolment Patients will be followed up until 2024	Safety and efficacy of the CD34+ transformed cells. Plasma levels of α-gal A Levels of α-gal A in blood leukocytes and bone marrow Transduction efficiency	The findings showed that all patients had near normal levels of α-gal A within 1 week. The vector was observed in blood leukocytes and bone marrow, and plasma. The study had no treatment related adverse events. Three patients were noted to have discontinued the enzyme therapy.
[45] Croci et al. (2020)	Characterisation of human neuronal model from Rett Syndrome (RTT) derived fibroblasts and iPSC-derived neurons	Four patients with classical RTT were selected All patients had the *MECP2* c.473C > T mutation	Gene editing CRISPR/Cas9 toolkit to correct the mutation in patient derived primary cells	The study demonstrated high homology directed repair editing efficiency of the toolkit in all four patients. The CRISPR/Cas9 method was able to correct the mutational hotspot (c.473 C > T) with high precision in primary cells derived from individuals with RTT. There was a negligible rate of indels confirming that the method was highly specific for the *MECP2* gene.
[46] Croci et al. (2020)	Gene editing in human RTT primary cells	Two patients with variant RTT carrying variants of the *FOXG1* gene Patient 1 (4-year-old male) had a missense variant c.688C > T Patient 2 (33-year-old female) had a nonsense variant c.765G > A	AAV mediated CRISPR/Cas9 targeting of *FOXG1* variants in patient derived fibroblasts, iPSCs and iPSC-derived neurons	The study demonstrated that AAV-CRISPR/Cas9 technology is able to successfully target and correct *FOXG1* variants in patient derived primary cells. The method was also able to preserve gene expression and normal protein levels and also show that infection using AAV was efficient in delivering the genetic material to patient derived iPSC-derived neurons.
[47] Andoh-Noda et al. (2015)	Exploration of pluripotent neuronal stem cells derived from individuals with RTT Patients were 10-year-old twins with a confirmed mutation in RTT	Fibroblasts were obtained from the twin girls with RTT to establish the pluripotent stem cells. Both girls had identical *MECP2* frame-shift mutations but showed different symptom severity	Expression patterns in patient derived fibroblasts Generation of pluripotent cell lines Global gene expression and assays in RTT stem cells and neuronal cells	The findings from RTT human induced pluripotent stem cells (iPSC) showed that MeCP2 is involved in the differentiation of neuronal cells. A reduction in MeCP2 accelerates the RTT stem cells towards the astrocyte lineage. It was suggested that the brains of Rett patients may have a larger number of astrocytes when compared to the neurotypical population.
[48] Adhikari et al. (2021)	Study evaluating an Angelman syndrome model using *Ube3a* expressing lentivector in haemopoietic stem cells to deliver functional UBE3A	Human CD34+ haemopoietic stem cells were obtained from umbilical cord blood	To assess the validity of the Angelman model Whether CD34+ human haemopoietic stem cells could be transduced to an animal model	The study demonstrated human CD34+ haemopoietic stem cells can successfully deliver a *Ube3a*-expressing lentiviral vector. Significant improvements were noted in motor and cognitive assessments within the neonate and adult animal model transduced with gene modified cells. The authors suggested that this approach offers a therapeutic strategy for Angelman syndrome that would not be dependent on a critical time window.
[49] Wolter et al. (2020)	Study exploring Cas9-mediated gene therapy for Angelman syndrome	Primary human neural progenitor-derived (phNPC) neurons from fetal brain tissue	To evaluate UBE3A expression and whether Cas9-mediated targeting of *SNORD115* genes can unsilence paternal *Ube3a* in differentiated phNPC neurons	The study demonstrated that Cas9 can unsilence paternal Ube3a in differentiated human neurons when targeted to Snord115 genes. In animal models, gene therapy vector can restore UBE3A function and in utero reactivation of *patUbe3a* can be useful in treating the symptoms associated with Angelman syndrome. The authors surmise that restoring *UBE3A* function during the embryonic and early post-natal period is predicted to be more effective than restoring it during the adult phase.
[50] Russell, et al. (2017)	Study safety and efficacy of AAV2 *RPE65* gene therapy in patients with inherited retinal dystrophy Patients (n = 31) were assigned randomly the intervention (n = 21) or control (n = 10) Those in the intervention group (n = 21) were aged (mean ± SD): 14.7 ± 11.8 years Those in the control group (n = 10) were aged (mean ± SD): 15.9 ± 9.5 years	Patients had a confirmed *RPE65* mutations The patient group was stratified by age; those <10 year and those ≥10 years Vector was given by subretinal injections One patient from each group withdrew before intervention leaving n = 20 receiving intervention and n = 9 in the control group	The primary endpoint was the change from baseline at 1 year in MLMT performance Secondary endpoint was other light and vision assessments Safety assessments included physical and ophthalmic examinations, laboratory assessments and adverse events	At 12 months, the MLMT score was 1.8 in the intervention group vs. 0.2. in the control group (*p* = 0.0013). No serious treatment emergent adverse events were reported. The overall findings from the study demonstrated that the AAV2 *RPE65* gene therapy can restore RPE65 enzymatic activity and improve vision in patients with inherited retinal dystrophy. This method could be applied to other rare inherited disorders of blindness.
[51] Bainbridge et al. (2015)	Study assessing the safety and efficacy of AAV gene therapy for functional replacement of the faulty gene *RPE65* in 12 patients with Leber’s Congenital Amaurosis Patients were assessed at baseline and at specified intervals over 3 years Patients were aged between 6 to 23 years	All patients had early onset and severe retinal dystrophy The vector was administered at two dose levels Gene therapy was done via sub-retinal injections	Parameters of vision such as visual acuity, contrast sensitivity, colour vision and spectral sensitivity at baseline and over a course of 3 years Incidence of ocular adverse events	There was a modest improvement with gene therapy in the treated area in comparison to untreated eye. In six patients, there were variable improvements in retinal sensitivity for up to 3 years. Improvements peaked between 6 and 12 months and declined thereafter. Ocular adverse events were deemed of mild intensity. The greatest responses to improvements were seen in older patients (17, 18 and 23 years of age).
[52] Ferrua et al. (2019)	Lentivirus haemopoietic stem cell gene therapy in eight children with Wiskott–Aldrich syndrome (WAS)	Children either were eligible if they had a *WAS* mutation or had no functional WAS protein Age on day of gene therapy for the 8 patients ranged from 1.1 to 12.4 years Median (range) follow-up was 3.6 years (0.5–5.6)	The primary outcome was safety and tolerability of the gene therapy product Efficacy endpoints included survival rates, engraftment of genetically corrected haemopoietic stem cells and expression of WAS protein	All patients demonstrated a robust engraftment of transduced cells and WAS protein expression. Improvement in immune function and platelet counts were observed as evidenced by normal T-cell function, reduction of infection and discontinuation of immunoglobulin therapy. Adverse events were most frequent during the first 6 months post gene therapy and were mainly infections. Questionnaire data showed that gene therapy significantly improved the quality of life for patients and their families. Variability in disease severity and age at treatment were some factors that could contribute to differences between studies.
[53] Hacein-Bey Abina et al. (2015)	Assessment of safety and efficacy of gene transduced haemopoietic stem cells in seven patients with Wiskott–Aldrich syndrome Patients were aged between 0.8 and 15.5 years (mean: 7 years) Follow-up period was between 9 and 42 months	All patients had a single infusion of lentivirus modified CD34^+^ cells	Primary outcome measures were improvements in eczema, frequency and severity of infections, bleeding, autoimmunity and number of hospitalisations Secondary outcome measures were those that focused on improvements in aspects related to immunology and haematology and lentivirus vector integration	The majority of patients (6/7) demonstrated clinical benefit following treatment. One patient died 7 months post treatment due to a pre-existing drug resistant infection. All patients were free of both haematological based treatments (blood products) and thrombopoietic agonists. After 2 years post-treatment, hospitalisation reduced from 25 to 0 days. Eczema was resolved in all affected patients. All six patients showed high levels of vector engraftment and WAS protein expression in myeloid cells.
[54] Mueller, et al. (2020)	Case study of two patients (patient 1: 22-year-old and patient 2: 56-year-old) with familial amyotrophic lateral sclerosis (ALS)	Both patients had mutations in their gene encoding for *SOD1* Patient 1 had a missense mutation (A5V) Patient 2 had a homozygous missense mutation (D91A)	Single intrathecal infusion of AAV encoding a microRNA targeting SOD1 The ALS Functional Rating Scale Revised was used to assess the clinical course Physiological measures were used to determine vital capacity and isometric limb strength	Meningoradiculitis developed in Patient 1 after the infusion but not in Patient 2 who was pre-treated with immunosuppressive drugs. Patient 1 showed short lived improvements in the strength of the right leg, however, it was unclear whether this remained stable or improved slightly post treatment. Post 15.6 months after the initiation of treatment and 20.5 months after ALS onset, Patient 1 died of a respiratory arrest. The authors suggest that AAV targeted gene suppression offers a way to provide a sustained effect using a single dose. However, this may also cause long-lasting side effects from using the AAV.
[55] Tabrizi et al. (2019)	Randomised, placebo-controlled double-blind study evaluating the safety and efficacy of an antisense oligonucleotide (HTT_Rx_) in patients with Huntington’s disease Active group (age (years) ± SD: 46 ± 10, n = 34) Placebo group (age (years) ± SD: 49 ± 10, n = 12)	Patients were eligible if they had early Huntington’s disease (36 or more CAG reports in *HTT* and clinical stage 1 disease There were five ascending dose cohorts of 10, 30, 60, 90, or 120 mg HTT_Rx_ was administered via an intrathecal bolus injection	Safety and efficacy following HTT_Rx_ treatment Secondary endpoints were pharmacokinetics of HTT_Rx_ in the CSF Exploratory endpoints included those clinical end points relevant in Huntington’s disease such as mutant HTT and ventricular volume	There were no serious treatment-related adverse events following intrathecal administration of HTT_Rx_. The findings also demonstrated a dose dependent decrease in the concentration of mutant HTT in the CSF. There were no differences in functional, cognitive, psychiatric and neurological clinical outcomes in patients who received placebo and patients that received active treatment at any dose level.

**Notes:** ^a^ Arsa-cel: gene therapy consisting of an autologous haemopoietic stem cell and progenitor cell population that has been transduced ex vivo with a lentivirus vector containing human arylsulfatase A. Abbreviations: Adrenoleukodystrophy (ALD); alpha-sarcoglycan (SGCA); alpha-galactosidase A (α-gal A); Amyotrophic lateral sclerosis (ALS); Adeno-associated virus (AAV); ARSA (human arylsulfatase A); CK (creatine kinase); ceroid lipofuscinosis 7 (CLN7); cerebrospinal fluid (CSF); Children’s Hospital of Philadelphia Infant Test of Neuromuscular Disorders (CHOP-INTEND); clustered regularly interspaced short palindromic repeats (CRISPR); compound muscle action potential (CMAP); CRISPR associated protein 9 (Cas9); Duchenne muscular dystrophy (DMD); Forkhead Box G1 (FOXG1); gross motor function measure (GMFM-88); huntingtin (HTT); induced Pluripotent Stem Cells (iPSC); limb-girdle muscular dystrophy type 2D (LGMD2D); magnetic resonance imaging (MRI); methyl CpG binding protein 2 (MECP2); metachromatic leukodystrophy (MLD); multi-luminance mobility testing (MLMT); N/A (not applicable); North Star Ambulatory Assessment (NSAA); primary human neural progenitor-derived cells (phNPC); Rett Syndrome (RTT); retinal pigment epithelium–specific protein 65 kDa (RPE65); six minute walk test (6MWT); N-sulfoglycosamine sulfohydrolase (SGSH); sulfatase-modifying factor (SUMF1); Spinal Muscular Atrophy Type 1 (SMA1); Standard Deviation (SD); survival motor neuron 1 (SMN1); small nucleolar RNA (SNORD); superoxide dismutase 1 (SOD1); Tay–Sachs disease (TSD); E3 ubiquitin-protein ligase (UBE3A); Wiskott–Aldrich syndrome (WAS).

**Table 2:** Disorders with the brain as the primary target—patterns in individuals with Rett Syndrome.

Disorder	Key Findings	Gene Therapy or Gene Editing Considerations for Individuals with Rett Syndrome (RTT)
Metachromatic Leukodystrophy (MLD)	Infants with MLD entering rapid disease progression did not benefit from treatment. The therapeutic window for intervention with best clinical outcome was early phase without rapid decline. Assessing baseline characteristics of cognitive and motor function helped predict the response to therapy.	Timing of gene therapy/gene editing in relation to disease progression is likely to be important. Treatment before the regression stage in RTT is likely to be beneficial. It is likely that those without much neurological/motor symptoms will benefit the most. We suggest that wearable sensor-based biomarkers of motor activity and autonomic function may be useful in stratifying patients when gene therapy/gene editing is trialled. A combined approach using wearable sensors alongside the MPSS, and other natural history data could help identify neurobehavioral profiles more or less likely to respond to gene therapy or gene editing, allowing for phenotype-based dosing strategies. Pharmacogenomics may identify pharmacogenomic profiles that predict response to gene therapy or gene editing. Pharmacogenomics would assist in minimising drug reactions from long-term immunosuppressant use and potential immune responses caused by viral vectors and transgene products.
Cerebral Adrenoleukodystrophy	Patients with relatively normal neurological status combined with early treatment respond better to gene therapy. There is lower uptake of functional genetic material of gene therapy (clinical vector copies) in patients who were the most functionally impaired.	This finding suggests that gene therapy or editing may not be as effective if there are already signs of neurological deterioration. The severity of the illness could impact the uptake of functional genetic material into individual cells.
Mucopolysaccharidosis Type IIIA (MPS Type IIIA)	In MPS Type IIIA disease, neurocognitive improvements were mainly observed in the youngest participant. Findings were mixed in the other three participants. It was suggested that gene therapy would be the most effective for children <5 years of age and without noticeable neurological deterioration.	Gene therapy/gene editing would probably be of most benefit during very early stages before regression occurs. Pre-symptomatic biomarkers would assist in identifying subtle changes before symptom onset.
Huntington’s disease	The genetic product used resulted in a dose-dependent decrease in mutant HTT protein in the CSF of patients with Huntington’s disease, but there was no functional change in neurological, psychiatric and cognitive outcomes. Treatment may not have reached the correct parts of the brain in patients with Huntington’s disease.	Intrathecal administration of genetic material may not lead to adequate penetration into the brain. Protein levels in the CSF do not accurately reflect protein activity levels in the brain. Extrapolating the biodistribution of delivery vehicles from pre-clinical models would not necessarily predict the biodistribution of genetic products in patients. Administration routes that cover the entire brain area or target specific areas might be more effective.
Tay–Sachs disease (TSD)	Combined thalamic and intrathecal delivery methods offer a conduit for global CNS biodistribution of AAV. The therapeutic effect could be augmented using combined delivery routes. Patients showed improvements after treatment such as myelination and clinical stabilization. The younger patient received half the dose of gene therapy.	Intracranial administration targeting specific brain regions may be more effective but riskier. If therapy could be initiated earlier, lower doses of genetic product could be used potentially reducing dose-dependent adverse events. Quantifying mosaics in different brain regions using molecular biomarkers helps to understand whether certain brain regions in RTT could be better exploited by gene therapy/editing. Novel gene editing technologies might help overcome the problems with mosaicism and dosage sensitivity.

Abbreviations: AAV (Adeno-Associated Virus); CNS (Central Nervous System); CSF (cerebrospinal fluid); MLD (Metachromatic Leukodystrophy); MPSS (Multi-system Profile of Symptoms Scale); MPS Type IIIA (Mucopolysaccharidosis Type IIIA); RTT (Rett Syndrome), TSD (Tay–Sachs disease).

## Data Availability

The data contained and used in this systematic was derived from following six databases: PubMed, Scopus, Cochrane, PsycINFO, Embase and Web of Science that are openly accessible in the public domain.

## Supplementary Materials

The following supporting information can be downloaded at: https://www.mdpi.com/article/10.3390/ijms24109023/s1.
