# Supplementary material for: Evidence Synthesis of Gene Therapy and Gene Editing from Different Disorders—Implications for Individuals with Rett Syndrome: A Systematic Review
Source: Int J Mol Sci. 2023 May 19;24(10):9023. doi: 10.3390/ijms24109023 (PMC10219055; doi:10.3390/ijms24109023)
Supplement: Supplementary file 1 [file ijms-24-09023-s001.zip › ijms-2327618-SI.pdf]

International Journal of
Molecular Sciences

MDPI

Supplementary Table S1: Summary of records using the secondary search terms

Search Terms	Databases Searched					
	**PubMed**	**Cochrane**	**Scopus**	**PsycINFO**	**Embase**	**Web of Science**
(Angelman Syndrome) AND (gene therapy *) AND (Brain *)	37	1	37	1	11	34
(MECP2 duplication syndrome OR MDS) AND (gene therapy *) AND (Brain *)	34	6	19	0	8	35
(Tuberous sclerosis OR tuberous sclerosis complex) AND (gene therapy *) AND (Brain *)	98	2	116	0	19	71
Number of article(s) identified	1	0	0	0	0	0

* Truncation symbol used in the searches ensured that all the different forms of the word were searched.
